# Antimicrobial Drug Resistance in *Escherichia coli* from Humans and Food Animals, United States, 1950–2002

**DOI:** 10.3201/eid1805.111153

**Published:** 2012-05

**Authors:** Daniel A. Tadesse, Shaohua Zhao, Emily Tong, Sherry Ayers, Aparna Singh, Mary J. Bartholomew, Patrick F. McDermott

**Affiliations:** Food and Drug Administration, Laurel, Maryland, USA (D.A. Tadesse, S. Zhao, E. Tong, S. Ayers, A. Singh, P.F. McDermott);; Food and Drug Administration, Rockville, Maryland, USA (M.J. Bartholomew)

**Keywords:** Escherichia coli, bacteria, antimicrobial drug resistance humans, food animals, United States

## Abstract

We conducted a retrospective study of *Escherichia coli* isolates recovered from human and food animal samples during 1950–2002 to assess historical changes in antimicrobial drug resistance. A total of 1,729 *E. coli* isolates (983 from humans, 323 from cattle, 138 from chickens, and 285 from pigs) were tested for susceptibility to 15 antimicrobial drugs. A significant upward trend in resistance was observed for ampicillin (p<0.001), sulfonamide (p<0.001), and tetracycline (p<0.001). Animal strains showed increased resistance to 11/15 antimicrobial agents, including ampicillin (p<0.001), sulfonamide (p<0.01), and gentamicin (p<0.001). Multidrug resistance (≥3 antimicrobial drug classes) in *E. coli* increased from 7.2% during the 1950s to 63.6% during the 2000s. The most frequent co-resistant phenotype observed was to tetracycline and streptomycin (29.7%), followed by tetracycline and sulfonamide (29.0%). These data describe the evolution of resistance after introduction of new antimicrobial agents into clinical medicine and help explain the range of resistance in modern *E. coli* isolates.

## Abstract 2

Determining drug resistance trends will optimize treatment and public health responses.

Antimicrobial drugs have played an indispensable role in decreasing illness and death associated with infectious diseases in animals and humans. However, selective pressure exerted by antimicrobial drug use also has been the major driving force behind the emergence and spread of drug-resistance traits among pathogenic and commensal bacteria (*1*). In addition, resistance has developed after advent of every major class of antimicrobial drugs, varying in time from as short as 1 year (penicillin) to >10 years (vancomycin) (*2**,**3*).

*Escherichia coli* is usually a commensal bacterium of humans and animals. Pathogenic variants cause intestinal and extraintestinal infections, including gastroenteritis, urinary tract infection, meningitis, peritonitis, and septicemia (*4**,**5*). Therapeutic options vary depending on the type of infection. For example, for urinary tract infections, trimethoprim/sulfamethoxazole and fluoroquinolones are treatments of choice (*6*), whereas for Shiga toxin–producing *E. coli* infections, antimicrobial drug therapy is not recommended (*7*). *E. coli* is sometimes used as a sentinel for monitoring antimicrobial drug resistance in fecal bacteria because it is found more frequently in a wide range of hosts, acquires resistance easily (*8*), and is a reliable indicator of resistance in salmonellae (*9*).

Surveillance data show that resistance in *E. coli* is consistently highest for antimicrobial agents that have been in use the longest time in human and veterinary medicine (*10*). The past 2 decades have witnessed major increases in emergence and spread of multidrug-resistant bacteria and increasing resistance to newer compounds, such as fluoroquinolones and certain cephalosporins (*3*). For example, a study of the susceptibility of *E. coli* isolates recovered from hospitals during a 12-year period (1971–1982) showed no major change in resistance to any of the antimicrobial drugs tested (*11*). In contrast, a retrospective analysis of *E. coli* from urine specimens collected from patients during 1997–2007 showed an increasing resistance trend for ciprofloxacin, trimethoprim/sulfamethoxazole, and amoxicillin/clavulanic acid (*12*). Similarly a 30-year (1979–2009) follow-up study on *E. coli* in Sweden showed an increasing resistance trend for ampicillin, sulfonamide, trimethoprim, and gentamicin (*13*). Although studies of farms have shown an association of multidrug-resistant *E. coli* with chronic antimicrobial drug exposure (*14**,**15*), there are few data on temporal trends of antimicrobial drug resistance in food animal *E. coli* isolates, particularly those recovered before 1980. Recent data are available in several countries that established resistance monitoring programs during the mid-1990s.

In the United States, the National Antimicrobial Resistance Monitoring System (NARMS) was established in 1996 to prospectively monitor changes in antimicrobial drug susceptibilities of zoonotic foodborne bacteria, including *E. coli* from retail meats (chicken breast, pork chops, ground beef, ground turkey), and chickens at slaughter. During 2000–2008, NARMS laboratories tested 13,521 *E. coli* isolates from chickens to determine the MIC to antimicrobial drugs essential in human and veterinary medicine. The resistance trend in chickens observed during this period varied on the basis of the antimicrobial agents. For example, resistance during 2000–2008 decreased slightly for kanamycin (16.1% to 10.2%), streptomycin (77.5% to 54.6%), trimethoprim/sulfamethoxazole (17.2% to 9.1%), and tetracycline (68.4% to 47.4%). Cefoxitin resistance increased from 7.4% in 2000 to 15% in 2006, and ceftriaxone resistance increased from 6.3% to 13.5%. Ciprofloxacin resistance remained low (<1%) during this period.

To better understand the historical emergence of resistance since the advent of the antimicrobial drug age, which led to baseline data in the first year of NARMS testing, we assayed *E. coli* collections from human and animal sources obtained during 1950–2002 for antimicrobial drug susceptibility. This information, when coupled with secular surveillance data, will provide a broader picture of evolution of resistance and lay the groundwork for understanding genetic mechanisms of resistance development and dissemination.

## Materials and Methods

### Bacterial Strains

A total of 1,729 *E. coli* isolates from human and animal samples obtained from different US states were used in this study. Isolates were obtained by the American Type Culture Collection (ATCC) (Manassas, VA, USA) from the *E. coli* Reference Center (ECRC) at Pennsylvania State University (University Park, PA, USA) and the Centers for Disease Control and Prevention (CDC) (Atlanta, GA, USA) under contract with the US Food and Drug Administration Center for Veterinary Medicine (Rockville, MD, USA). These isolates were recovered from human and animal specimens (e.g., feces, blood, kidney, lymph nodes, urine, cerebrospinal fluid, peritoneal fluid, pleural fluid) submitted to ECRC and CDC from state public health and veterinary diagnostic laboratories. For human isolates obtained from CDC, most acquired during 1948 through the late 1980s were maintained on trypticase soy agar stabs sealed with paraffin and stored at room temperature. Starting in the late 1980s, strains were frozen in trypticase soy broth containing 30% glycerol at −70°C. Similarly, isolates were stored, according to the ECRC standard protocol at −70°C to −80°C in trypticase soy broth containing 30% glycerol until further processing.

Of 1,729 *E. coli* isolates, 983 (56.8%) were recovered from humans during 1950–2001, and the remaining 746 (43.2%) were recovered from animals during 1962–2002. Three hundred twenty-three (43.2%) *E. coli* isolates of animal origin were from cattle, 138 (18.5%) from chickens, and 285 (38.2%) from pigs. Fifty percent were from 10 states: Pennsylvania (183, 11.2%), California (100, 5.8%), Ohio (90, 5.2%), Maryland (83, 4.8%), Minnesota (82, 4.7%), Illinois (72, 4.2%), Texas (72, 4.2%), New York (70, 4.1%), North Carolina (61, 3.5%), and Virginia (58, 3.4%). Distribution of isolates by source and year are shown in Table 1.

**Table 1:** Period of isolation and source of *Escherichia coli* isolates tested, United States, 1950–2002

Period	Source, no. isolates			
	Human	Cattle	Chickens	Pigs
1950–1959	180	0	0	0
1960–1969	112	17	0	15
1970–1979	292	32	11	25
1980–1989	211	86	65	96
1990–1999	182	108	44	81
2000–2002	6	81	18	68

### Antimicrobial Drug Susceptibility Testing

Each isolate was streaked on trypticase soy agar supplemented with 5% defibrinated sheep blood (Becton Dickinson, Sparks, MD, USA) before antimicrobial drug susceptibility testing. MICs were determined by using the Sensititer automated antimicrobial susceptibility system (Trek Diagnostic Systems, Cleveland, OH, USA) according to the manufacturer’s instructions. Results were interpreted according to National Committee for Clinical and Laboratory Standards criteria (*16*) where available (Table 2). Antimicrobial drugs tested were ampicillin, amoxicillin/clavulanic acid, cefoxitin, ceftiofur, cephalothin, ceftriaxone, ciprofloxacin, nalidixic acid, streptomycin, gentamicin, kanamycin, chloramphenicol, tetracycline, sulfonamide, and trimethoprim/sulfamethoxazole. *E. coli* ATCC 25922 and ATCC 35218, *Enterococcus faecalis* ATCC 29212, *Staphylococcus aureus* ATCC 29213, and *Pseudomonas aeruginosa* ATCC 27853 were used as quality control organisms in MIC determinations. Multidrug resistance was defined as resistance to >3 classes of antimicrobial drugs.

**Table 2:** Antimicrobial drug resistance phenotypes of *Escherichia coli* isolates from different sources, United States, 1950–2002*

Drug class	Drug	Resistance breakpoint, µg/mL	% Resistance					Timeline for clinical use of drugs (reference)
			Overall, n = 1,729	Human, n = 983	Cattle, n = 323	Chickens, n = 138	Pigs, n = 285	
Penicillins	AMP	>32	24.1	16.5	35	34.1	33.3	1961 (*17*)
β-lactam/β-lactamase inhibitor combinations	AUG	>32	5.6	2.4	12.7	7.3	7.4	1984 (*17*)
Cephems	CEP	>32	12.9	8.8	20.1	12.3	19.3	1964 (*18*)
	FOX	>32	4.4	1.5	9.3	5.1	8.4	1977 (*19*)
	TIO	>8	2.3	0.1	7.4	1.5	4.2	1988 (FDA Green Book)†
	AXO	>4	2.4	0.1	7.7	1.5	4.6	1984 (FDA Orange Book)‡
Phenicols	CHL	>32	8.1	3.7	18	8.7	11.9	1947 (*17*)
Aminoglycosides	GEN	>16	6.7	0.1	16.1	16.7	14	1963 (*17*)
	KAN	>64	19.3	5.7	39.9	29.7	37.5	1957 (*17*)
	STR§	>64	34.2	15.3	61.3	58	57.2	1943 (*17*)
Quinolones	CIP	>4	0.4	0	1.9	0.7	0	1987 (*17*)
	NAL	>32	1.7	1	3.7	1.5	1.8	1962 (*19*)
Tetracyclines	TET	>16	40.9	18	71.2	68.8	72.3	1948 (*17*)
Folate pathway inhibitors	SUL	>512	36.2	19.9	61	60.9	52.6	1936 (*19*)
	TMP/SMX	>4	7	2.2	16.1	13.8	9.8	1968 (*19*)

*AMP, ampicillin; AUG, amoxicillin/clavulanic acid; CEP, cephalothin; FOX, cefoxitin; TIO, ceftiofur; FDA, Food and Drug Administration; AXO, ceftriaxone; CHL, chloramphenicol; GEN, gentamicin; KAN, kanamycin; STR, streptomycin; CIP, ciprofloxacin; NAL, nalidixic acid; TET, tetracycline; SUL, sulfonamide; TMP/SMX, trimethoprim/sulfamethoxazole. †www.fda.gov/AnimalVeterinary/Products/ApprovedAnimalDrugProducts ‡www.fda.gov/drugs/developmentapprovalprocess/ucm079068.htm §No Clinical and Laboratory Standards Institute breakpoint. The National Antimicrobials Resistance Monitoring System breakpoint was used.

### Statistical Analysis

The Mann-Kendall test, a nonparametric statistical test, was performed to detect a monotone increasing or decreasing resistance trend over time. Magnitude of annual change was estimated by using a slope parameter, Q, and the Sen nonparametric method (*20*). Calculations were performed by using the Excel (Microsoft, Redmond, WA, USA) template Mann-Kendall test for trend and Sen slope estimates (*21*). For time series <10 annual percentage resistance values, significance of the trend was determined from the exact distribution of the S test statistic, and a normal approximation (z statistic) was used when there were >10 values. Significance was assessed at 4 levels (α = 0.001, 0.01, 0.05, and 0.1); p values <0.05 were considered significant. Comparisons of drug resistance profiles between different sources (human, cattle, chicken, and pigs) were conducted by using the χ^2^ test; p values <0.05 was considered significant.

## Results

### Antimicrobial Drug Susceptibility

Overall, 934 (54.0%) of 1,729 *E. coli* were resistant to >1 antimicrobial drug. As expected, the most common resistance phenotypes were to older drugs such as tetracycline (40.9%) (introduced in 1948), sulfonamide (36.2%) (introduced in 1936), streptomycin (34.2%) (introduced in 1943), and ampicillin (24.1%) (introduced in 1961). A much smaller number of isolates were resistant to antimicrobial drugs introduced for clinical use since 1980, such as amoxicillin/clavulanic acid (5.6%) (introduced in 1984), ceftriaxone (2.4%) (introduced in 1984), ceftiofur (2.3%) (introduced in 1988), and ciprofloxacin (0.4%) (introduced in 1987) (Table 2).

When analyzed by source, *E. coli* isolates of animal origin were more resistant than those of human origin. Among 983 human isolates, resistance was observed most often to sulfonamide (19.9%), followed by tetracycline (18%) and ampicillin (16.5%). No human *E. coli* isolates showed resistance to ciprofloxacin, and 1 isolate (0.1%) from 1997 showed resistance to ceftiofur, ceftriaxone, and gentamicin. Of 746 isolates recovered from animal sources, 531 (71.1%) were resistant to tetracycline, 441 (59%) to streptomycin, 431 (57.7%) to sulfonamide, 277 (37.1%) to kanamycin, and 255 (34.1%) to ampicillin. Among animal *E. coli* isolates, the rate of resistance was significantly higher in cattle isolates than in pig isolates for chloramphenicol (p = 0.039), amoxicillin/clavulanic acid (p = 0.03), sulfonamide (p = 0.038), and trimethoprim/sulfamethoxazole (p = 0.022). There was a significant difference in resistance rate between cattle and chicken isolates to ceftiofur (p = 0.008), ceftriaxone (p = 0.008), chloramphenicol (p = 0.011), and kanamycin (p = 0.037) (Table 2).

**Figure 1:**
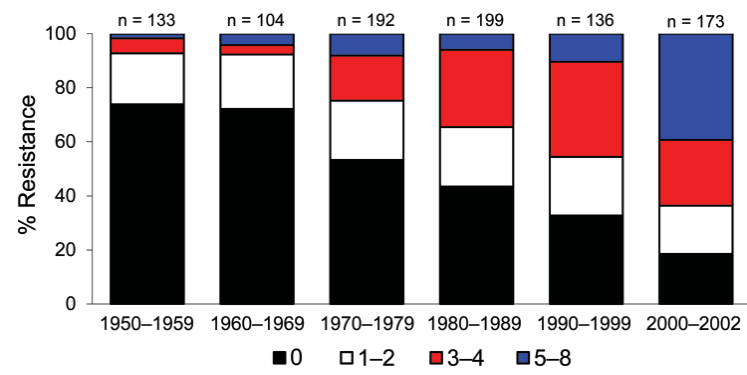
Change in antimicrobial drug resistance patterns among *Escherichia coli* isolates, United States, 1950–2002.

**Figure 2:**
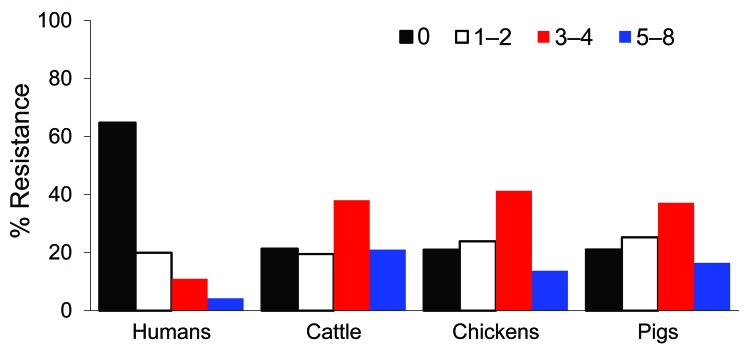
Distribution of multidrug resistance patterns among *Escherichia coli* isolates recovered from different sources, United States, 1950–2002.

Antimicrobial drug resistance was observed for drugs tested at different frequencies (Table 2). Seven hundred ninety-six (46%) *E. coli* isolates analyzed were susceptible to all 15 drugs tested. Among these pan-susceptible isolates, 637 (80%) were from humans, 69 (8.7%) from cattle, 60 (7.5%) from pigs, and 29 (3.6%) from chickens. Approximately 65% of human isolates were pan susceptible, compared with ≈20% of cattle, chicken, and pig isolates (Table 3). The proportion of pan-susceptible *E. coli* isolates decreased from 73.9% in 1950–1959 to 18.5% in 2000–2002. Conversely, multidrug resistance increased from 7.2% in 1950–1959 to 63.6% in 2000–2002 (Figure 1). Five hundred seventy (32.9%) *E. coli* isolates showed multidrug-resistant phenotypes, and 176 (10.2%) showed resistance to >5 drug classes. A larger proportion of multidrug-resistant isolates was recovered from animals than humans (Figure 2). One hundred ninety-one (59.1%) isolates from cattle, 153 (53.7%) from pigs, and 76 (55.1%) from chickens were resistant to >3 drug classes (Table 3), compared with 15.3% from humans. Two strains showed resistance to all 15 drugs tested; both strains were recovered from cattle in 2001.

**Table 3:** Multiple antimicrobial drug resistance among *Escherichia coli* isolates analyzed by source, United States, 1950–2002

No. drug classes to which isolates are resistant	% Resistant				
	Human, n = 983	Animal			Total, n = 1,729
		Cattle, n = 323	Chickens, n =138	Pigs, n = 285	
0*	64.8	21.4	21.0	21.1	46
1	12.4	9	7.2	9.1	10.8
2	7.5	10.5	16.7	16.1	10.2
3	6.5	22.6	26.8	23.9	14
4	4.5	15.5	14.5	13.3	8.8
5	3.8	7.7	7.2	8.4	5.5
6	0.4	6.2	4.3	2.8	2.2
7	0.1	5.3	2.2	5.3	2.1
8	0	1.9	0	0	0.3

*Susceptible to all drug classes.

Concurrent resistance to tetracycline and streptomycin was the most common co-resistance phenotype (29.7%), followed by resistance to tetracycline and sulfonamide (29.0%); tetracycline, sulfonamide, and streptomycin (23.9%); tetracycline and ampicillin (18.8%); and tetracycline, ampicillin, streptomycin, and sulfonamide (12.9%). A total of 130 (92.9%) of 140 chloramphenicol-resistant *E. coli* isolates were also resistant to tetracycline. Resistance to ceftriaxone, ceftiofur, and ciprofloxacin was rare and found only in isolates resistant to ≥7 drugs. More than 80% of these isolates were resistant to ampicillin, amoxicillin/clavulanic acid, cephalothin, cefoxitin, streptomycin, tetracycline, and sulfonamide.

### Antimicrobial Drug Resistance Trends

**Figure 3:**
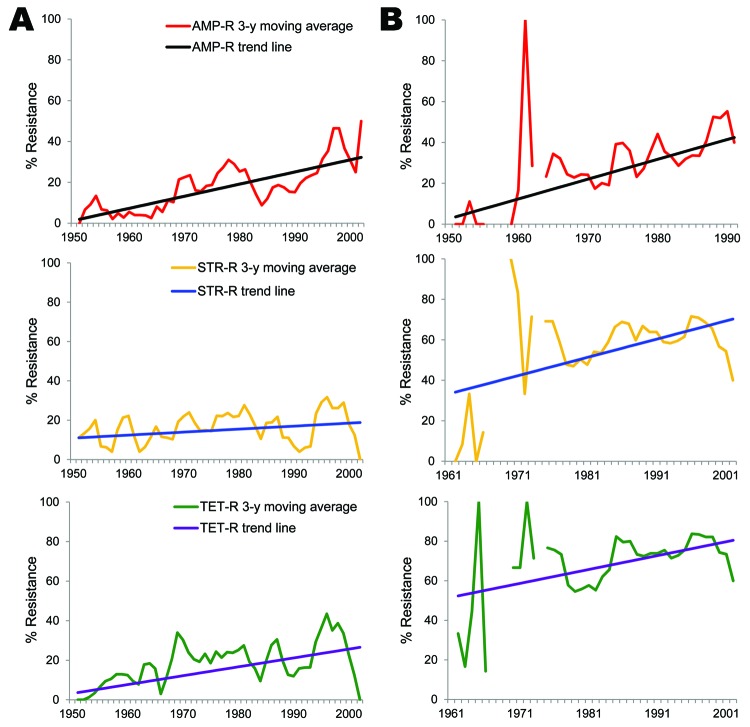
Trend analysis of selected antimicrobial agents among *Escherichia coli* isolates from humans (A) and animals (B), United States, 1950–2002. AMP-R, ampicillin resistance; STR-R, streptomycin resistance; TET-R, tetracycline resistance.

The major goal of this study was to document antimicrobial drug resistance among historical bacteria from humans and animals to associate emergence of resistance with approval of new antimicrobial classes. Animal *E. coli* isolates showed an increasing resistance trend to 11 antimicrobial agents (ampicillin, sulfonamide, tetracycline, cephalothin, trimethoprim/sulfamethoxazole, streptomycin, chloramphenicol, cefoxitin, gentamicin, amoxicillin/clavulanic acid, and kanamycin), and human *E. coli* isolates showed an increasing trend in resistance only to ampicillin, sulfonamide, and tetracycline (Figure 3, Table 4).

**Table 4:** Mann-Kendall and Sen estimates for trend statistics of antimicrobial drug resistance among *Escherichia coli* isolates of human and animal origin, United States, 1950–2002

Drug	Source	Years of time series	No.*	Mann-Kendall trend test Z value	Sen’s slope estimate	
					p value	Q† (95% CI)
Ampicillin	Human	1950–2001	52	4.91	<0.001	0.59 (0.38–0.81)
	Animal	1962–2002	37	3.95	<0.001	0.97 (0.60–1.33)
Cephalothrin	Human	1950–2001	52	−2.38	<0.05	−0.20 (−0.34 to 0)
	Animal	1962–2002	37	2.52	<0.05	0.43 (0.08–0.77)
Sulfonamide	Human	1950–2001	52	3.41	<0.001	0.49 (0.23–0.73)
	Animal	1962–2002	37	3.24	<0.01	1.11 (0.34–1.85)
Streptomycin	Human	1950–2001	52	1.16	>0.1	0.15 (−0.10 to 0.39)
	Animal	1962–2002	37	2.24	<0.05	0.90 (0.14–1.41)
Tetracycline	Human	1950–2001	52	3.84	<0.001	0.45 (0.22–0.70)
	Animal	1962–2002	37	2.78	<0.01	0.70 (0.21–1.21)
Trimethoprim/sulfamethoxazole	Animal	1982–2002	21	2.64	<0.01	1.17 (0.32–2.15)
Chloramphenicol	Animal	1962–2002	37	2.75	<0.01	0.30 (0–0.59)
Cefoxitin	Animal	1982–2002	21	3.72	<0.001	0.88 (0.28–1.38)
Gentamicin	Animal	1978–2002	25	4.53	<0.001	1.28 (0.90–1.69)
Amoxicillin/clavulanic acid	Animal	1962–2002	37	3.09	<0.01	0.14 (0–0.38)
Kanamycin	Animal	1962–2002	7	4.38	<0.001	1.11 (0.59–1.50)

*Number of annual values for calculation of trend analysis. †Magnitude of annual percentage increase or decrease.

Human *E. coli* isolates showed an increased resistance trend for ampicillin (0.59%/year, 95% CI 0.38%–0.81%; p<0.001), sulfonamide (0.49%/year, 95% CI 0.23%–0.73%; p<0.001), and tetracycline (0.45%/year, 95% CI 0.22%–0.70%; p<0.001), and this trend ranged during the study period from 0% to 66.7% for ampicillin, 0% to 50% for sulfonamide, and 0% to 58% for tetracycline (Table 4). The resistance rate to ampicillin in animal *E. coli* isolates was similar to that in human isolates and ranged from 0% to 69.4%. In contrast, resistance rates for sulfonamide and tetracycline among animal *E. coli* isolates ranged from 0% to 73.7% and 0% to 85.5%, respectively, and were higher than those for human isolates. There was a linear increasing trend in resistance to ampicillin (0.97%/year, 95% CI 0.60%–1.33%; p<0.001), sulfonamide (1.11%/year, 95% CI 0.34%–1.85%; p<0.01), and tetracycline (0.7%/year, 95% CI 0.21%–1.21%; p<0.01) (Table 4).

Cephalothin resistance significantly increased over time (0.43%/year, 95% CI 0.08%–0.77%; p<0.05) among animal *E. coli* isolates. In contrast, a decreasing resistance trend to cephalothin (−0.2%/year, 95% CI −0.34% to 0%; p<0.05) was observed among human isolates (Table 4). Although animal *E. coli* isolates showed an increasing rate of streptomycin resistance over time (0.9%/year, 95% CI 0.14%–1.41%; p<0.05), there was no significant increase among human *E. coli* isolates during the study period (0.15%/year, 95% CI −0.10% to 0.39%; p>0.1) (Table 4). In our study isolates, gentamicin resistance was observed in the 1980s among animal *E. coli* isolates but not until the late 1990s in human isolates. In animal *E. coli* isolates, the prevalence of gentamicin resistance increased from 0% during the 1970s to 28.1% during 2000–2002, and an increasing trend for resistance to gentamicin (1.28%/year, 95% CI 0.90%–1.69%; p<0.001) was first observed in the 1980s and reached a prevalence of 40% in 2002. Chloramphenicol resistance varied widely between human and animal isolates (yearly range 0%–46.8% among animal isolates and 0%–20% among human isolates). A linear increase in chloramphenicol resistance was observed among animal isolates (p<0.01), which increased 0.30%/year. Ceftiofur-resistant and ceftriaxone-resistant strains were not detected until 1990–1999 among human and animal *E. coli* isolates. None of the human *E. coli* isolates showed resistance to ciprofloxacin.

There was no monotonic resistance trend for trimethoprim/sulfamethoxazole, chloramphenicol, cefoxitin, ceftiofur, ceftriaxone, gentamicin, amoxicillin/clavulanic acid, nalidixic acid, and kanamycin among human *E. coli* isolates. Similarly, animal *E. coli* isolates did not show a monotonic resistance trend for ceftiofur, ceftriaxone, ciprofloxacin, and nalidixic acid.

## Discussion

To help characterize evolution of drug resistance in *E. coli* since antimicrobial drugs were first widely used, we tested existing strain collections of *E. coli* for their susceptibility to a common panel of 15 antimicrobial agents. We tested 1,729 *E. coli* isolates from human and animal sources for susceptibility trends during the past 6 decades.

Resistance to sulfonamide was one of the most common resistance profiles identified among our study isolates and showed a monotone increasing resistance trend over time. Sulfonamide resistance has been observed in human *E. coli* isolates since 1950 and in animal isolates since 1964. Sulfonamides were introduced in the 1930s and have been in continuous use for >70 years. These drugs were administered alone from the 1930s through the 1960s in humans and were almost exclusively combined with diaminopyrimidines (e.g., trimethoprim) since the 1970s. In animal production systems, SUL is one of the most commonly used drugs as a single agent or in combination with diaminopyrimidines (e.g., ormetoprim) (*14*). A high prevalence of clinical resistance to sulfonamides was reported in enteric bacteria isolated from healthy food animals and humans (*10**,**22**,**23*) and is often associated with acquisition of the resistance genes *sul1* and *sul2* (*23*).

Sulfonamide resistance genes are commonly associated with mobile genetic elements, and these elements play a major role in dissemination of multiple antimicrobial drug resistance genes in *E. coli* isolates (*24**–**26*). In addition, despite a major reduction in the rate of sulfonamide use in the United Kingdom in 1995, resistance to sulfonamides persisted at high rates among clinical *E. coli* isolates (*22**,**25*). Similarly, a 30-year (1979–2009) follow-up study on antimicrobial drug resistance at the Karolinska Hospital in Stockholm, Sweden, reported an increase in sulfonamide resistance despite decreased use (*13*).

Linkage of sulfonamide resistance genes, particularly as a constituent of class I integrons, to determinants conferring resistance to antimicrobial drugs that are still commonly used might help explain persistence of sulfonamide resistance (*22*). In our study, 80% (502/627) and 74% (462/627) of sulfonamide-resistant *E. coli* isolates were also resistant to tetracycline and streptomycin, respectively. Wu et al. (*27*) demonstrated that streptomycin and ampicillin are the 2 most frequently co-transferred resistance phenotypes among sulfonamide-resistant *E. coli* isolates recovered from pigs, pig carcasses, and humans. In addition to co-selection by drugs still commonly used, Enne et al. (*28*) and Bean et al. (*25*) suggested that lack of selective disadvantage of *sul2* (the most prevalent determinant of sulfonamide resistance) carriage and the genetic mobility of *sul2* might account for persistence in the absence of clinical selection pressure.

Tetracycline resistance was the most common type of resistance observed and the most prevalent resistance phenotype in animal isolates (71.1%). This finding is not surprising because tetracycline has been widely used in therapy and to promote feed efficiency in animal production systems since its approval in 1948 (*2**,**14*). Persistence of tetracycline resistance was reported in animal coliforms a decade after it was no longer used in feed or for treatment (*29*). We commonly found co-resistance for tetracycline with streptomycin, sulfonamide, ampicillin, and chloramphenicol, as in other studies (*23**,**30**,**31*).

A small percentage of *E. coli* showed resistance to chloramphenicol, a drug approved in 1947 for human clinical use. Chloramphenicol is not approved for use in food animals in the United States. Persistence of chloramphenicol resistance in *E. coli* has been observed by other authors (*10**,**32*). Florfenicol, a closely related drug, was approved for treatment of respiratory diseases in cattle in the United States in 1996. Florfenicol resistance mediated by the *flo* gene confers nonenzymatic cross-resistance to chloramphenicol (*33**,**34*) and might select for nascent resistance in recent strains. Of known chloramphenicol-resistance genes, only a small number mediate resistance to florfenicol (*34*). For example, chloramphenicol-resistant strains in which resistance is exclusively based on activity of chloramphenicol acetyltransferases do not show resistance to florfenicol (*35*). Of 104 chloramphenicol-resistant animal *E. coli* isolates, 35.6% were isolated before approval of florfenicol. More than 90% of chloramphenicol-resistant *E. coli* isolates were concurrently resistant to tetracycline. In addition, our data showed not only persistence of chloramphenicol but an increasing tetracycline and SUL resistance trend over time among animal *E. coli* isolates. These observations could be explained by co-selection of mobile resistance elements or by unknown substrate(s) for the chloramphenicol-resistance determinants that serve as a selection pressure (*23**,**36*).

Gentamicin was approved for use in 1963 (*2*). Although gentamicin resistance was rare in human *E. coli* isolates, we found resistance rates <40% among animal *E. coli* in 2002. Since 1980, resistance to gentamicin has increased among animal *E. coli* isolates. The overall rate of gentamicin resistance was slightly higher in chicken (16.6%) and cattle (16%) isolates than in pig (14%) isolates. Gentamicin is widely used in the poultry industry. Aminoglycosides approved for use in food animals in the United States include dihydostreptomycin, efrotomycin, hygromycin B, neomycin, spectinomycin, streptomycin, and apramycin (*37*). A correlation between use of apramycin at the farm level and apramycin/gentamycin–resistant *E. coli* in diseased pigs and healthy finishers was reported (*15*). Yates et al. (*38*) reported apramycin-resistant *E. coli* isolates that were resistant to gentamicin and tobramycin, which are drugs used in human medicine. In our study, 93% of gentamicin-resistant *E. coli* isolates were multidrug resistant (>3 classes of drugs). Eighty-one percent (94/116) were resistant to >5 antimicrobial drugs, including 95.7% (111/116) to streptomycin, 93.1% (108/116) to sulfonamides, and 91.4% (106/116) to tetracycline.

Our data showed lack of a monotonic trend for extended-spectrum cephalosporins resistance. Ceftiofur, a third-generation cephalosporin, was first approved in 1988 for veterinary use in food animals to treat a variety of gram-negative bacterial infections, including acute bovine respiratory diseases (*39*). In our culture collection, ceftiofur resistance was not detected before 1993 in animal isolates and before 1997 in human isolates. In NARMS *E. coli* collections, ceftiofur resistance was detected in the first years of testing among chicken carcasses (6.3% in 2000) and retail chicken breast samples (7.1% in 2002) (*10*). Studies have shown ceftiofur use in animals can select for extended-spectrum cephalosporin resistance, including ceftriaxone resistance in bacteria isolated from animals and humans (*40*).

In the present study, 1 human *E. coli* isolate recovered in 1997 showed resistance to ceftiofur and ceftriaxone. This isolate was also resistant to 9 other antimicrobial drugs. Studies on *E. coli* isolates with decreased susceptibilities to ceftiofur and ceftriaxone showed carriage of a *bla*_CMY_ allele that conferred resistance to cephalothin, ampicillin, and amoxicillin/clavulanic acid, as in salmonellae (*24**,**40*). Additional data that include more years are needed to determine the resistance trend over time because third-generation cephalosporins were introduced in the 1980s.

A recent NARMS report showed that resistance to ceftriaxone ranged from 6.3% to 13.5% among *E. coli* isolates from chickens during 2000–2008; resistance to ceftiofur ranged from 4.4% to 10.5% during the same period (*10*). Currently, the molecular mechanisms of antimicrobial drug resistance development and evolution of these resistance genes over time are being investigated.

Our study has limitations because of its retrospective nature and reliance on preexisting culture collections for analysis. These limitations resulted in an uneven distribution of isolates per year and decade, incomplete or absent patient/host information regarding prior treatment history, and potential for bias in selecting isolates that were ultimately tested in this study. ECRC and CDC accept clinical samples for diagnostic purposes. Thus, isolate sets cannot be considered truly random. Also, patient information was limited; we had no data for prior antimicrobial drug exposure, travel, and other epidemiologic information. Therefore, analyses of resistance as a function of time were confounded. We selected the nonparametric tests of Mann-Kendall and Sen for trend analysis because they are suitable for non-normally distributed data and data with small number of observations.

Despite these limitations, this analysis provides foundational information for resistance development over time, laying the groundwork for understanding evolution of multidrug resistance at the genetic level. In addition, these data show that multidrug resistance is not a congenital feature of *E. coli*, and that drug use and resistance are closely related temporally. Work is ongoing to analyze this isolate set for alleles underlying resistance and compare them with recent isolates. This work will provide more definitive data on how resistance gene clusters have evolved and the context in which genes are maintained in the absence of known selection pressures.
